# Surgical repair of facial nerve defects by an autologous great auricular nerve transplantation: a retrospective, 12-month, follow-up study

**DOI:** 10.3389/froh.2026.1920749

**Published:** 2026-10-05

**Authors:** Huawei Liu, Qi Ning, Xiaodan Mu, Wenting Bi, Qingyan Sun, Zhigang Cai, Shijun Li, Xiaofeng Shan, Min Hu

**Affiliations:** 1 Department of Stomatology, The First Medical Center of PLA General Hospital, Beijing, China; 2 Department of Stomatology, Beijing Friendship Hospital Affiliated to Capital Medical University, Beijing, China; 3 Department of Stomatology, Beijing Hospital of Integrated Traditional Chinese and Western Medicine, Beijing, China; 4 Department of Oral surgery, Peking University School and Hospital of Stomatology, Beijing, China; 5 National Center of Stomatology, National Clinical Research Center for Oral Diseases, Beijing, China; 6 National Engineering Research Center of Oral Biomaterials and Digital Medical Devices, Beijing, China; 7 First Clinical Division, Peking University School and Hospital of Stomatology, Beijing, China

**Keywords:** facial nerve defects, follow-up, function recovery, great auricular nerve, House–Brackmann grading system, nerve transplantation

## Abstract

**Background:**

To observe facial nerve function recovery from an autologous great auricular nerve (GAN) transplantation in repairing facial nerve defects.

**Methods:**

Thirty-six patients were included. Facial nerve functions were evaluated by using the House–Brackmann grading system. Abnormal sensations after the GAN sacrifice were recorded.

**Results:**

The follow-up lasted for an average of 18.5 (12–60) months. Facial nerve defects were caused by parotid gland malignancies (*n* = 30) or trauma (*n* = 6), with an average length of 2.6 ± 1.1 (1.0–6.5) cm. One case showed tumor recurrence during the follow-up. Lack of feeling (78.1%) was the dominant postoperative abnormal sensation, followed by lack of sensitivity (53.1%) and abnormal sensation (40.6%). Long facial nerve defects (>3 cm) and preoperative facial paralysis combined with parotid gland malignancies retarded facial nerve function recovery.

**Conclusion:**

Autologous GAN transplantation is effective for repairing facial nerve defects and recovering facial nerve function during the 12-month follow-up period.

## 1 Introduction

Facial nerve defects can be caused by maxillofacial trauma, resection of parotid gland tumors, and iatrogenic injury and may lead to facial paralysis, atrophy of facial expression muscles, and keratitis (1–3). Surgical repair and reconstruction, especially minimally invasive surgeries, bring maximal benefits to the recovery of facial appearance and movement (4, 5). Encouragingly, microsurgical reconstruction methods are burgeoning, such as facial–hypoglossal nerve anastomosis, transfacial nerve transplantation, free transplantation of the greater auricular nerve (GAN), superficial cervical nerve, thoracodorsal nerve, and sural nerve, pedicled transplantation, and vascularized transplantation (6–8).

However, surgical repair of facial nerve defects is rendered challenging by a local anatomy propped up with complex branches and interconnections. For facial nerve grafting, in addition, potential damage to donor nerves induces a series of complications. The GAN divides into multiple branches that can appear in the surgical scope of the parotid gland, presenting a similar size to that of the facial nerve. The optimal location, size, and length make the GAN an ideal donor nerve for repairing facial nerve defects, but the long-term outcomes of GAN-based surgical repair are scant. In the present study, we retrospectively analyzed efficacy and safety during a 12-month follow-up after surgical repair of facial nerve defects with an autologous GAN transplantation.

## 2 Materials and methods

### 2.1 Clinical data of participants

This was a retrospective study involving 36 patients with facial nerve defects caused by parotid gland malignancies or maxillofacial trauma conducted between January 2011 and December 2020. All defects were surgically repaired via a parotid incision and immediately reconstructed by an autologous GAN transplantation through a one-stage procedure. Enrolled patients had a median age of 44.0 ± 15.7 (15–79) years and were followed up for an average of 18.5 ± 11.1 (12–60) months.

All the 36 patients with facial nerve defects caused by parotid gland malignancies or maxillofacial trauma were continuously selected from January 2011 to December 2020 in our institution. Excluded were those with previous ipsilateral facial surgeries, prior head and neck radiotherapy, severe diabetes mellitus or peripheral neuropathy, and follow-up < 12 months or lost to follow-up. Eligible patients, regardless of the parotid malignancy group (*n* = 30) or the trauma group (*n* = 6), were immediately managed by one-stage repair of facial nerve defects and GAN transplantation. To eliminate the potential confounders, baseline characteristics (e.g., age, gender, and underlying diseases) were compared between groups, without reporting significant differences. However, subgroup analyses based on smoking and other variables were not conducted because of a small sample size. A flowchart of patient selection is provided in Figure 1.

**Figure 1:**
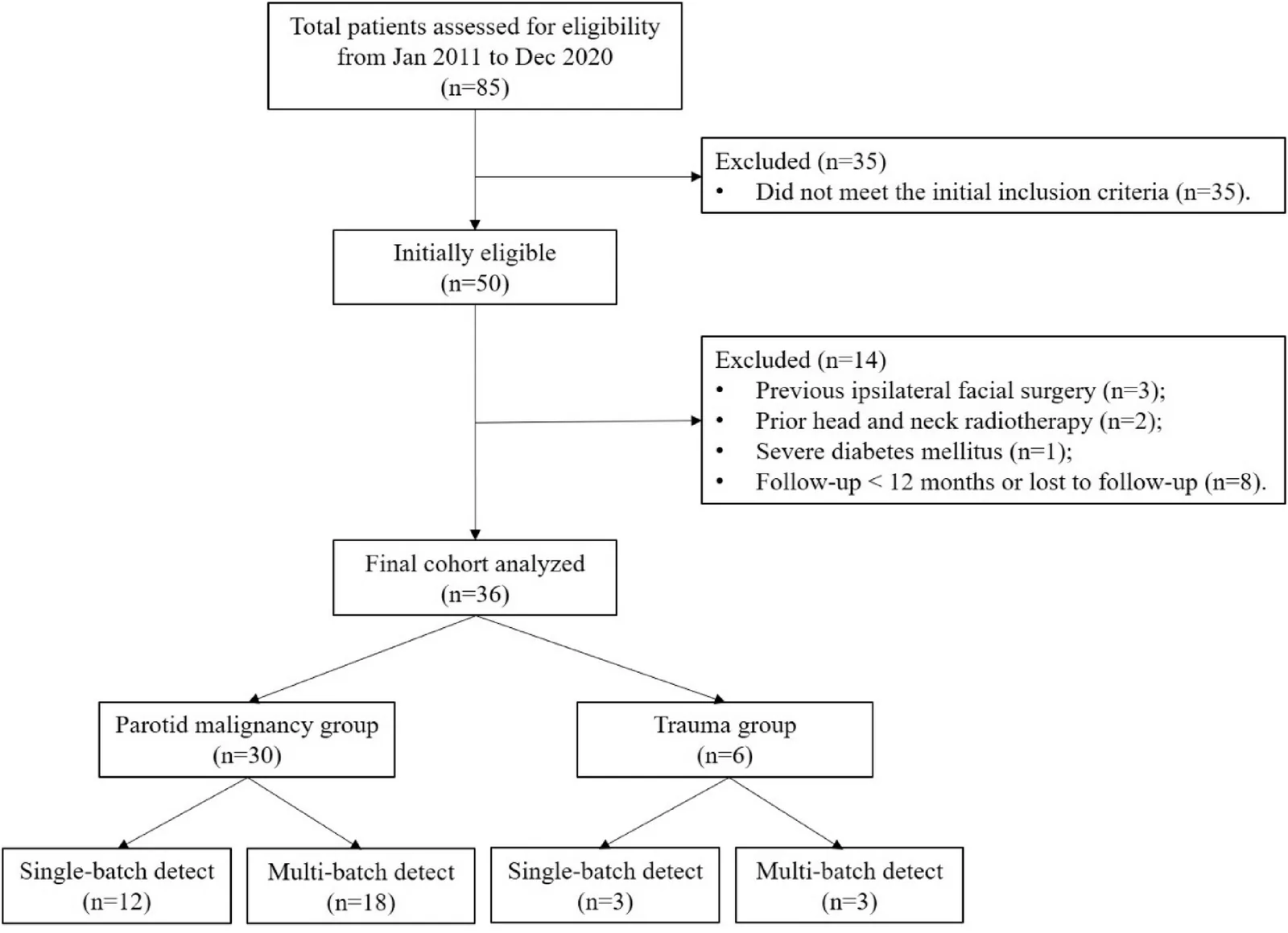
A flowchart of patient selection.

### 2.2 Surgical procedures

Patients with facial nerve defects due to parotid gland malignancies were surgically treated with total parotidectomy or superficial parotidectomy, followed by a resection of the involved facial nerve branches and a reconstruction of facial nerves using the GAN (Figure 2). Facial nerve injury caused by maxillofacial trauma is often accompanied by soft tissue damage and jawbone fractures. In these patients, facial nerve transplantation and anastomosis, as well as jaw bone fracture reduction and fixation, were performed simultaneously in one surgery, reducing the number of surgeries and trauma for patients (Figure 3).

**Figure 2:**
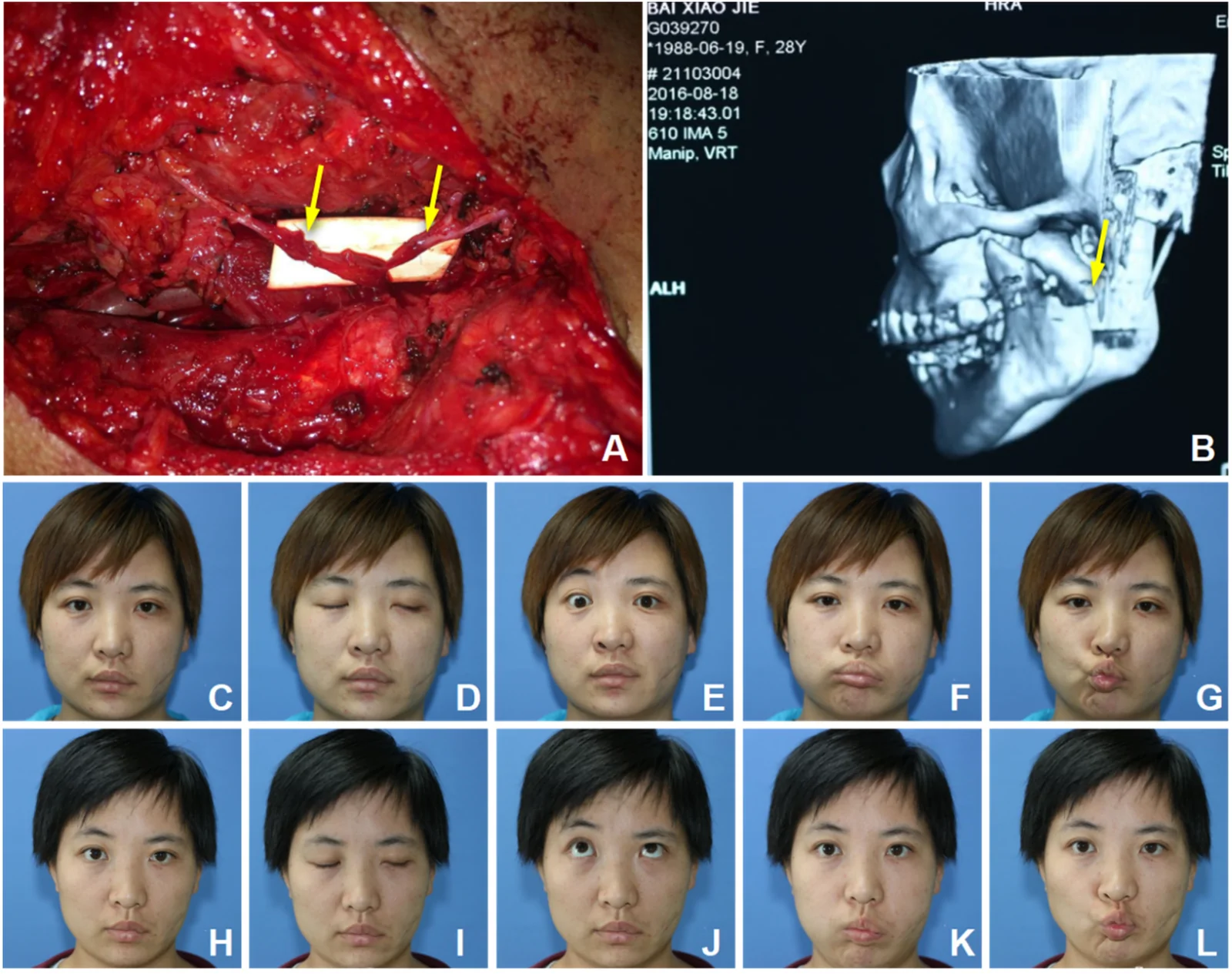
Representative images of a 28-year-old female patient with trauma-induced multibranch facial nerve defects repaired by an autologous GAN transplantation. **(A)** Multibranch facial nerve defects in the trunk, and cervical and temporal branches. (The yellow arrow indicates the anastomosis between the auricular nerve and the facial nerve for the autologous nerve transplantation.) **(B)** A CT image visualizing the left condyle fracture. A postoperative recovery of facial nerve function at 6 months in the static, facial nerve function HB classification reaches grade IV **(C)**, eyes closed **(D)**, eyebrows raised **(E)**, puffed cheek **(F)**, and lip pursing status **(G)**. A postoperative recovery of facial nerve function at 12 months in the static, facial nerve function HB classification reaches grade Ⅲ **(H)**, eyes closed **(I)**, eyebrows raised **(J)**, puffed cheek **(K)**, and lip pursing status **(L)** The patient's facial nerve function recovered from (**C–G**) HB Grade IV at 6 months postoperatively to (**H**–**L**) HB Grade III at 12 months postoperatively, demonstrating a progressive functional recovery beyond the 6-month assessment.

**Figure 3:**
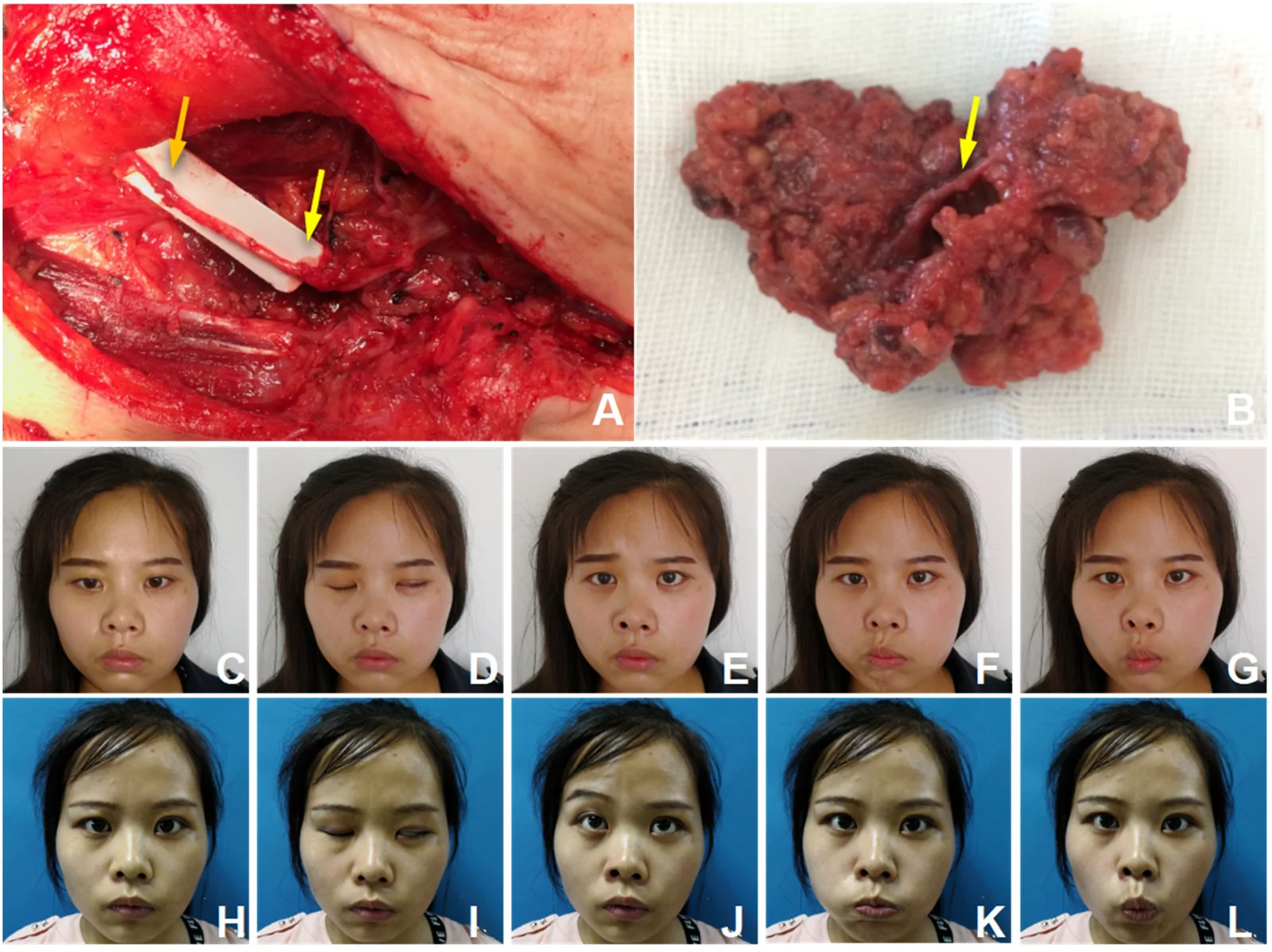
Representative images of a 34-year-old female patient with a single facial nerve defect caused by the left parotid acinar cell carcinoma and repaired by an autologous GAN transplantation. **(A)** A single-branch defect of the mandibular marginal branch of the facial nerve. (The yellow arrow indicates the anastomosis between the auricular nerve and the facial nerve.) **(B)** A tumor invades the mandibular marginal branch of the facial nerve. (The yellow arrow indicates that the nerve was invaded and wrapped by the tumor.) A postoperative recovery of facial nerve function at 6 months in the static, facial nerve function HB classification reaches grade Ⅳ **(C)**, eyes closed **(D)**, eyebrows raised **(E)**, puffed cheek **(F),** and lip pursing status. **(G)**. A postoperative recovery of facial nerve function at 12 months in the static, facial nerve function HB classification reaches grade Ⅲ **(H)**, eyes closed **(I)**, eyebrows raised **(J)**, puffed cheek **(K),** and lip pursing status **(L)**.

Briefly, branches of the GAN were carefully dissected and protected during the operation. The GAN measuring 1 cm longer than the facial nerve defect was harvested. If branches of the GAN were insufficient to repair all the facial nerve defects, important branches of the facial nerve (e.g., the zygomatic branch and mandibular marginal branch) were prioritized to be repaired. A selective repair strategy was adopted in cases with insufficient GAN branches to repair all defected facial nerve branches. The priority in repairing facial nerve branches was based on the order of their functional importance. The zygomatic branch that controls eyelid closure and protects the cornea was the prior branch to be repaired, followed by the marginal mandibular branch (controlling lower lip movement and oral corner symmetry), the buccal branch (controlling nasolabial groove morphology and oral corner movement), and then the remaining branches. If the initially harvested GAN segment was insufficient in the length or branch count to cover all facial nerve defects, the dissection was extended proximally along the GAN toward the cervical plexus. This additional dissection made GAN transplantation using a longer length or more branching segments to better match the specific requirements of the facial nerve defect (Figure 4). The proximal and distal ends of the facial nerve and the GAN were repaired and anastomosed with 9–0 nylon microsurgery suture under a double binocular surgical microscope.

**Figure 4:**
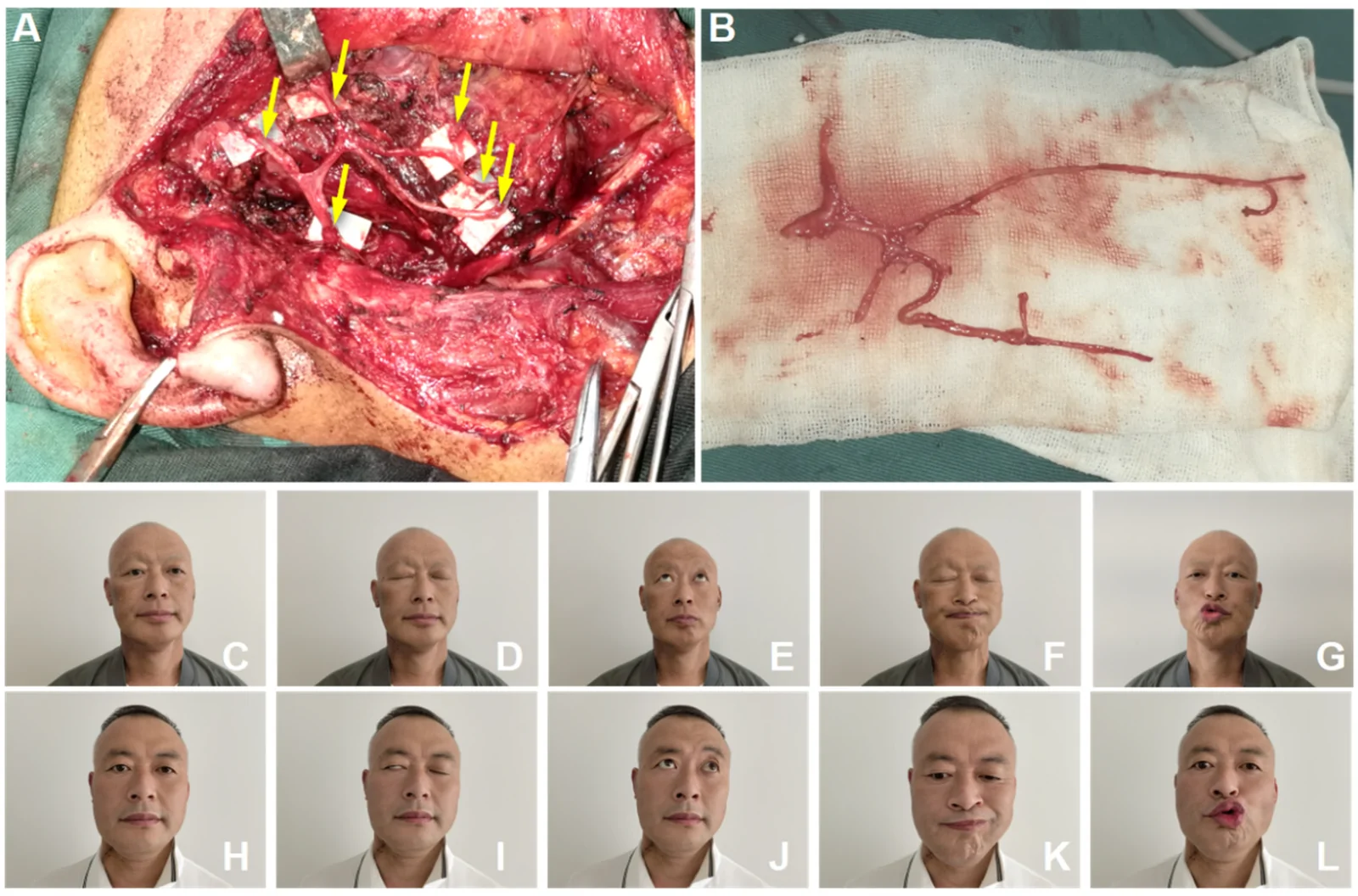
Representative images of a 51-year-old male with defects of all branches of the facial nerve caused by the right parotid duct carcinomas. He was surgically repaired with the greater auricular nerve and an autologous GAN transplantation. (**A**) Repair of defects in the total trunk and major branches of the facial nerve by an autologous auricular nerve transplantation. **(B)** An intraoperative preparation of the great auricular nerve. A postoperative recovery of facial nerve function at 6 months in the static, facial nerve function HB classification reaches grade IV **(C)**, eyes closed **(D)**, eyebrows raised **(E)**, puffed cheek **(F),** and lip pursing status **(G)**. A postoperative recovery of facial nerve function at 12 months in the static, facial nerve function HB classification reaches grade III **(H)**, eyes closed **(I)**, eyebrows raised **(J)**, puffed cheek **(K)**, and lip pursing status (**L**).

For patients without preoperative facial nerve paralysis but with tight adhesions between the malignancy and the facial nerve that could not be separated during the surgery, resection of these affected facial nerve segments and immediate reconstruction using the GAN was a necessary procedure to ensure the complete malignancy resection.

### 2.3 Follow-up

All patients were postoperatively followed up for an average of 18.5 ± 11.1 (12–60) months. The facial nerve function was evaluated by using the House–Brackmann grading system (9, 10), and tumor recurrences were recorded. Sensations in the following areas specific to the GAN sacrifice at 6 and 12 months postoperatively were assessed using an 7-item scale as previously reported: the anterior superior helix, posterior superior helix, lobule, concha, preauricular area, infraauricular area, and postauricular area (11). In addition, the frequency, duration, anxiety, extension, interference to daily activities, and worry about abnormal sensations experienced after the GAN sacrifice were quantified (11).

### 2.4 The House–Brackmann grading system

The facial nerve function of all patients was assessed by using the House–Brackmann grading system independently by two experienced senior physicians who had not been previously engaged in surgical procedures. They were pretrained with a validated consistency (Kappa > 0.8) and blinded to clinical information and surgical regimens of each case. The assessment was primarily conducted based on standardized preoperative and postoperative facial photographs and videos both in static and dynamic statues. Generally, the House–Brackmann grading system reflects an overall facial nerve function.

### 2.5 Statistical analysis

Data were expressed as median, range, and *n* (%), if appropriate. The Chi-square test was conducted to compare differences, and a significant difference was determined by *p* < 0.05. Influencing factors for the postoperative recovery of facial nerve function were screened by the univariate analysis and determined by using the Chi-square test or Fisher's exact test, with the postoperative House–Brackmann grading (acceptable recovery of Grade I–Ⅲ vs. poor recovery of Grade Ⅳ–Ⅵ) as the dependent variable, and the defect length (>3 cm vs. ≤3 cm), number of branches (single vs. multiple), and presence of preoperative facial paralysis (yes vs. no) as independent variables. Multivariate analysis was not further conducted because of the small sample size. Statistical analysis was performed using SPSS 22.0.

## 3 Results

### 3.1 Baseline characteristics

There were 30 patients suffering from facial nerve defects caused by parotid gland malignancies, including 8 cases of mucoepidermoid carcinomas (MECs), 12 of adenoid cystic carcinomas (ACCs), 3 of malignant pleomorphic adenomas (MPAs), 4 of acinar cell carcinomas (ACICs), and 3 of parotid duct carcinomas (PDCs). Single-branch defects were detected in 12 patients, and multibranch defects were found in 18 patients. Intraoperative frozen sections showed no residual tumor in the facial nerve stump. Trauma explained the cause of facial nerve defects in 6 patients, involving 3 with single-branch defects and 3 with multibranch defects. Overall, facial nerve defects measured an average of 2.6 ± 1.1 (1.0–6.5) cm (Table 1).

**Table 1:** Population data, etiology of facial nerve lesions, defect length, and functional outcomes.

S.No.	Age/sex	Etiology of facial nerve lesions	Follow-up time (months)	Defect length (cm)	Number of nerve branches (single or multiple)	Preoperation	Postoperation
						(HB) grade	(HB) grade
1	15/M	Tumor/MEC	60	6.5	Multiple	Ⅴ	Ⅴ
2	21/F	Tumor/ACC	12	2.2	Multiple	Ⅳ	Ⅴ
3	65/F	Tumor/MEC	18	4.	Multiple	Ⅲ	Ⅳ
4	29/F	Tumor/ACC	12	4.2	Multiple	Ⅱ	Ⅴ
5	31/M	Tumor/MPA	12	4.3	Multiple	Ⅲ	Ⅳ
6	52/M	Tumor/ACIC	12	2.5	Multiple	Ⅱ	Ⅳ
7	37/M	Tumor/ACC	12	3.1	Multiple	Ⅳ	Ⅴ
8	38/M	Tumor/ACIC	24	4	Multiple	Ⅱ	Ⅳ
9	38/F	Tumor/MEC	12	3.4	Multiple	Ⅲ	Ⅲ
10	34/M	Tumor/ACC	24	3.7	Single	Ⅲ	Ⅳ
11	34/F	Tumor/ACIC	12	2.5	Single	Ⅱ	Ⅳ
12	63/M	Tumor/MPA	18	2.3	Multiple	Ⅳ	Ⅴ
13	38/M	Tumor/ACIC	48	3.1	Single	Ⅳ	Ⅳ
14	38/F	Tumor/ACC	24	2.9	Multiple	Ⅱ	Ⅲ
15	40/M	Tumor/MEC	12	2.6	Multiple	Ⅰ	Ⅳ
16	79/F	Tumor/MEC	36	2.5	Single	Ⅰ	Ⅳ
17	45/F	Tumor/ACC	24	2.4	Multiple	Ⅰ	Ⅳ
18	47/F	Tumor/MEC	48	2.3	Single	Ⅰ	Ⅳ
19	72/M	Tumor/MEC	18	2.2	Single	Ⅰ	Ⅳ
20	51/M	Tumor/PDC	12	2	Multiple	Ⅰ	Ⅲ
21	34/F	Tumor/ACC	12	2	Single	Ⅰ	Ⅱ
22	56/M	Tumor/ACC	12	2.1	Multiple	Ⅰ	Ⅲ
23	57/F	Tumor/PDC	12	2	Single	Ⅰ	Ⅲ
24	59/M	Tumor/PDC	12	1.9	Multiple	Ⅰ	Ⅳ
25	37/F	Tumor/ACC	12	1.5	Multiple	Ⅰ	Ⅲ
26	26/M	Tumor/MEC	18	1.2	Single	Ⅰ	Ⅲ
27	66/M	Tumor/ACC	12	1.8	Single	Ⅰ	Ⅲ
28	71/F	Tumor/MPA	12	1	Single	Ⅰ	Ⅱ
29	49/M	Tumor/ACC	12	1.6	Multiple	Ⅰ	Ⅳ
30	44/M	Tumor/ACC	18	1.7	Multiple	Ⅰ	Ⅳ
31	24/F	Injury	12	3.3	Multiple	–	Ⅴ
32	30/M	Injury	12	2.2	Multiple	–	Ⅳ
33	43/M	Injury	12	1.5	Single	–	Ⅲ
34	62/M	Injury	24	3.5	Single	–	Ⅳ
35	28/F	Injury	24	2.2	Multiple	–	Ⅳ
36	32/M	Injury	12	1.2	Single	–	Ⅲ

### 3.2 Follow-up data

During the follow-up period, 1 case of tumor recurrence was reported and then controlled by 60 Gy radiotherapy. Preoperatively, there were 1 case of Grade V, 4 cases of Grade IV, 4 cases of Grade III;, 5 cases of Grade II, and 16 cases of Grade I among 30 patients with facial nerve defects caused by parotid gland malignancies. Postoperatively, there were 5 cases of Grade V, 15 cases of Grade IV, 8 cases of Grade III, and 2 cases of Grade II. The six patients with trauma-induced facial nerve defects, according to postoperative facial nerve function, were classified into Grade V (*n* = 1), Grade IV (*n* = 2), and Grade III (*n* = 3) (Table 1). Among the 12 patients who had available HB assessment results at both 6 and 12 months, 8 (66.7%) patients showed disease condition improvement, and 4 remained stable. No patient showed deterioration during this interval.

In the area innervated by the GAN, lack of feeling (78.1%) was the dominant abnormal sensation, followed by lack of sensitivity (53.1%) and abnormal sensation (40.6%) (Table 2). Abnormal sensations after the GAN sacrifice did not exert a significant effect on the quality of life (Table 3).

**Table 2:** Type of sensation perceived in the auricle and periauricular areas according to the Patel et al. (11) questionnaire.

Type of sensation	Number (%)
Stinging	9 (28.1%)
Abnormal sensation	13 (40.6%)
Burning	3 (9.4%)
Lack of feeling	25 (78.1%)
Pain	6 (18.8%)
Lack of sensitivity	17 (53.1%)
Discomfort	12 (37.5%)
Hypersensitivity	9 (28.1%)

According to the questionnaire by Patel et al., “lack of feeling” was defined as complete numbness (anesthesia) in the innervated area, whereas “lack of sensitivity” was defined as diminished but not absent sensation (hypoesthesia). These represent distinct degrees of sensory deficit following GAN sacrifice.

**Table 3:** Feelings experienced by patients according to the Patel et al. questionnaire.

Type of sensation	Range	Median (IQR)
Frequency	0–6	4 (3–6)
Length	0–6	6 (0–6)
Anxiety	0–6	1 (0–3)
Extension	0–4	4 (1–4)
Interference to daily routine	0–6	0 (0–1)
Worry about these abnormal feelings	0–6	0 (0–1)

### 3.3 Factors influencing the postoperative recovery of facial nerve function

Single-branch or multibranch defects did not influence the postoperative outcome of facial nerve function. Conversely, facial nerve defects longer than 3 cm, and preoperative facial paralysis combined with parotid gland malignancies, significantly influenced the postoperative facial nerve function improved by the autologous GAN transplantation (*P* < 0.05, Table 4).

**Table 4:** Single or multiple facial nerve defect, length of the facial nerve defect, and preoperative facial paralysis on the functional recovery of the facial nerve.

	Case number	Outcomes		*P*-value
		(HB) Grade I–Ⅲ	(HB) Grade Ⅳ–Ⅵ	
Facial nerve branches				0.1515^a^
Single	15	7	8	
Multiple	21	5	16	
Defect length (cm)				0.0407^b^
>3	11	1	10	
≤3	25	11	14	
Preoperative facial paralysis for tumors				0.0072^b^
Yes	14	2	12	
No	16	10	6	

a Chi-square tests. *P* > 0.05 suggested that the statistical difference was not significant.

b Chi-square tests. *P* < 0.05 suggested that the statistical difference was significant.

## 4 Discussion

Facial nerve defects are frequently caused by maxillofacial trauma and tumor resection, and occasionally by iatrogenic injury. Available approaches to repair facial nerve defects include direct facial nerve anastomosis, facial nerve transfer, autologous nerve transplantation, and nerve allograft transplantation (8, 12, 13). Generally, small facial nerve defects can be repaired by local free tension reduction and end-to-end anastomosis. An increased tension generated between the both ends of the facial nerve stump, however, obstructs a direct anastomosis in large facial nerve defects longer than 5 mm. Facial nerve transfer aims to anastomose the distal end of the facial nerve with the proximal end of other cranial nerves, such as the hypoglossal nerve, accessory nerve, and masseter nerve. Korte et al. for the first time reported the excellent outcome of an end-to-end anastomosis of hypoglossal–facial nerve transfer in restoring the facial nerve function (14). Nevertheless, the risk of hypoglossal nerve dysfunction of the donor side causes a negative impact on the quality of life.

The GAN, superficial cervical nerve, thoracodorsal nerve, and sural nerve are common donor nerves used in free autogenous nerve transplantation. With the advancement of medical techniques and materials, the success rate of autogenous nerve grafting has been increasing (4, 8, 15). The GAN is a promising candidate to be sacrificed to repair facial nerve defects (16–20). It is the largest cutaneous branch of the cervical plexus and provides sensory innervation to the skin on the parotid gland and the mastoid process and parts of the outer ear. Originating from the second and third cervical nerves (C2 and C3), the GAN divides into the anterior and posterior branches at the parotid gland (16, 21). The length and diameter of the GAN vary in previous studies, probably attributed to different measurements (22, 23). Interestingly, the median diameter of the GAN is close to that of the facial nerve, with a positive correlation between them. The anatomic superiority positions the GAN as a favorable option for the repair and reconstruction of facial nerve defects.

Notably, complications of the donor site injury after GAN sacrifice, such as the loss of sensory nerve in the innervated area, barely affect the postoperative quality of life (24, 25). In a 5-year follow-up study, postoperative sensations and 1-year quality of life were comparable in patients with parotid gland tumor either with the preservation or non-preservation of the GAN (26). In the present study, lack of feeling (78.1%) and lack of sensitivity (53.1%) were the dominant abnormal sensations in the innervated area after sacrificing the GAN. However, they did not affect the 1-year quality of life. Patients with facial nerve defects repaired by an autologous GAN transplantation were more concerned about tumor recurrence and recovery of facial paralysis.

The length of facial nerve defects and preoperative facial paralysis in patients with parotid gland malignancies were important factors affecting the postoperative recovery of facial nerve function. Prasad et al. reported an acceptable postoperative recovery of facial nerve defects within 4 cm by surgical repair, with most of them reaching a Grade Ⅲ House–Brackmann score (27). Consistent in our study, more patients with facial nerve defects shorter than 3 cm obtained postoperative Grade Ⅰ–Ⅲ nerve function recovery, better than that in those with larger defects, proving that the length of facial nerve defects is of great significance in the functional recovery of the autologous GAN transplantation. In addition to the above-mentioned factors, the timing of functional assessment itself plays a critical role. Our observations suggest that facial nerve function continues to improve beyond 6 months post-transplantation, with the majority of patients showing further gains at the 12-month assessment. This finding underscores the importance of adequate follow-up duration when evaluating the final functional outcome of nerve grafting.

Preoperative facial paralysis significantly influences the postoperative recovery of facial nerve function in patients with facial nerve defects caused by parotid gland malignancies (28–30). Okochi et al. reported that the duration of the preoperative paralysis sustains and correlates with the degree of postoperative facial nerve dysfunction (30). In this study, the proportion of patients reaching a moderate improvement of facial nerve function (Grade Ⅰ–Ⅲ) after the autologous GAN transplantation was significantly higher in those without preoperative facial paralysis, compared with those with it. Parotid gland malignancies invade the facial nerve axon, leading to the denervation of targeted muscles, and insufficient nutritional supply to facial neurons. Muscle atrophy and weakness can be irreversible after 6 months of facial nerve damage due to a deteriorated intramuscular nerve sheath and loss of supportive satellite cells, eventually harming the postoperative functional recovery after nerve repair and reconstruction (6). Hontanilla et al. reported that the functional recovery of facial nerve defects repaired by nerve transplantation was not influenced by radiotherapy (31). In the present study, 12 patients with ACC treated with intraoperative radioactive seed implantation all achieved a postoperative recovery of facial nerve function.

Five major extracranial branches originate from the facial nerve. For the repair and regeneration of axons in multibranch defects, lacking an effective direction guidance at the facial nerve bifurcation results in the generation of nerve balls and failure of nerve defect repair. Here, an autologous GAN transplantation allowed a similar functional recovery between single and multibranch facial nerve defects, which could be attributed to the ability of autologous nerve transplantation to guide regenerating axons in the correct direction toward their target muscles.

The limitations of this study should be noted. First, it was a single-center retrospective study with a small sample size and lacking a control group treated with other nerve grafts or direct nerve anastomosis. Whether GAN transplantation in repairing facial nerve defects is superior to other techniques should be further validated in prospective randomized controlled trials. Second, the exact molecular mechanism of an autologous GAN transplantation in repairing facial nerve defects needs to be further explored. Third, abnormal sensations in the donor site were mainly assessed within 12 months postoperatively, and continuous sensory function monitoring data for patients with a longer follow-up period were scant. Whether abnormal sensations influenced the long-term quality of life should be further explored in a prolonged follow-up study. Fourth, the selective repair strategy adopted in our study may objectively improve the overall House–Brackmann grading system. A refined regional facial nerve function evaluation system can be used in future studies to more accurately evaluate the recovery of different facial nerve branches. Finally, this study was a single-center retrospective study with a limited sample size (*n* = 36) that allowed only a univariate analysis to identify influencing factors. The limited sample size may have resulted in insufficient statistical power to detect some potential associations. Therefore, our results should be regarded only as exploratory findings and need to be confirmed by large-scale studies.

## 5 Conclusion

Autologous GAN transplantation is an effective approach to repair facial nerve defects and can enable an acceptable recovery of facial nerve function during the 12-month follow-up period.

## Data Availability

The original contributions presented in the study are included in the article/Supplementary Material; further inquiries can be directed to the corresponding author.
